# Development of a Statistical Shape Model and Assessment of Anatomical Shape Variations in the Hemipelvis

**DOI:** 10.3390/jcm12113767

**Published:** 2023-05-30

**Authors:** Willemina A. van Veldhuizen, Hylke van der Wel, Hennie Y. Kuipers, Joep Kraeima, Kaj ten Duis, Jelmer M. Wolterink, Jean-Paul P. M. de Vries, Richte C. L. Schuurmann, Frank F. A. IJpma

**Affiliations:** 1 Department of Surgery, University Medical Center Groningen, 9713 GZ Groningen, The Netherlands; 2 Department of Oral and Maxillofacial Surgery/3D Lab, University Medical Center Groningen, 9713 GZ Groningen, The Netherlands; 3 Department of Applied Mathematics, Technical Medical Centre, 7500 AE Enschede, The Netherlands; 4 Multimodality Medical Imaging Group, Technical Medical Centre, University of Twente, 7500 AE Enschede, The Netherlands

**Keywords:** pelvis, pelvic fracture, statistical shape modeling, principal component analysis, osteosynthesis, 3D geometrical model

## Abstract

Knowledge about anatomical shape variations in the pelvis is mandatory for selection, fitting, positioning, and fixation in pelvic surgery. The current knowledge on pelvic shape variation mostly relies on point-to-point measurements on 2D X-ray images and computed tomography (CT) slices. Three-dimensional region-specific assessments of pelvic morphology are scarce. Our aim was to develop a statistical shape model of the hemipelvis to assess anatomical shape variations in the hemipelvis. CT scans of 200 patients (100 male and 100 female) were used to obtain segmentations. An iterative closest point algorithm was performed to register these 3D segmentations, so a principal component analysis (PCA) could be performed, and a statistical shape model (SSM) of the hemipelvis was developed. The first 15 principal components (PCs) described 90% of the total shape variation, and the reconstruction ability of this SSM resulted in a root mean square error of 1.58 (95% CI: 1.53–1.63) mm. In summary, an SSM of the hemipelvis was developed, which describes the shape variations in a Caucasian population and is able to reconstruct an aberrant hemipelvis. Principal component analyses demonstrated that, in a general population, anatomical shape variations were mostly related to differences in the size of the pelvis (e.g., PC1 describes 68% of the total shape variation, which is attributed to size). Differences between the male and female pelvis were most pronounced in the iliac wing and pubic rami regions. These regions are often subject to injuries. Future clinical applications of our newly developed SSM may be relevant for SSM-based semi-automatic virtual reconstruction of a fractured hemipelvis as part of preoperative planning. Lastly, for companies, using our SSM might be interesting in order to assess which sizes of pelvic implants should be produced to provide proper-fitting implants for most of the population.

## 1. Introduction

Acetabular fractures are among the most challenging injuries to treat in orthopedic trauma care. Fractures often have complex patterns and multiple fragments, which are displaced in several directions [1]. The three-dimensional (3D) complexity of acetabular fractures, in combination with anatomical shape variations in the pelvis, poses a specific challenge to achieving optimal fracture reduction and the fitting, positioning, and fixation of osteosynthesis plates.

Traditionally, the evaluation of acetabular fractures and surgical planning was based on 2D X-ray images and 2D computed tomography (CT) slices. More recently, 3D volume reconstructions of CT data are being used for preoperative planning [2,3]. A virtual model of the fractured pelvis can be used for proper implant selection, the pre-modeling of conventional plates using a 3D printed template, and eventually patient-specific implant design [4,5,6]. A virtual model can be obtained by mirroring the contralateral hemipelvis. If this contralateral hemipelvis is not available, due to a bilateral fracture or an in situ implant in the contralateral hemipelvis, a statistical shape model (SSM) may be of added value to create a patient-specific virtual reconstruction for preoperative plate fitting based on the patient’s sex and body length [7,8].

An SSM represents the average shape of many 3D objects, which is created via the correspondence mapping of all objects. A principal component analysis (PCA) is the main method for constructing such an SSM [9]. The resulting principal components (PCs) sequentially encode the most dominant features that explain the shape variation in an object. SSMs have been previously applied for different body regions [10,11,12]. The current knowledge about pelvic shape variation consists mostly of plate fitting, virtual reconstructions, and asymmetry evaluations [7,8,10,13,14,15,16,17,18]. The basic anatomical shape variation in the whole lower extremity in a Caucasian population has only been assessed in the research by Audenaert et al. (2019), recommending a dataset of at least 200 segmentations [10]. However, 3D region-specific knowledge about how the male and female pelvis differs is still scarce [19,20]. Therefore, our study adds to this by providing an in-depth analysis of the anatomical shape variation in the hemipelvis based on a dataset with a balanced and sufficient number of male and female patients. The results will be clinically relevant for the preoperative planning of pelvic fracture surgery (i.e., for virtual reconstructions in the case of bilateral injury to the pelvis and eventually for implant development).

The aim of this study was to develop a statistical shape model of the hemipelvis to investigate variations in pelvic morphology. The second objective was to define which anatomical regions of the hemipelvis differ between male and female hemipelves.

## 2. Materials and Methods

### 2.1. Study Population

A diagnostic imaging study was performed to develop a statistical shape model of the hemipelvis. Non-contrast-enhanced CT scans of patients treated at the surgical department of the University Medical Center Groningen (Groningen, the Netherlands) between 2012 and 2020 were retrieved from the picture archiving and communication system. These patients received a CT scan for an abdominal surgical procedure that was not related to the pelvic bone. The inclusion criteria were the availability of a CT scan; age > 18 years; the presence of an intact left hemipelvis; and the availability of demographic information, including age, sex, height, and weight. Moreover, the complete pelvis needed to be present on the available CT scan. The exclusion criteria were (a history of) pelvic fractures, congenital hip defects, and pelvic implants in situ. The first 100 male and the first 100 female patients from this consecutive dataset, who met these criteria, were included in our study. The inclusion of the required number of patients from this dataset was finalized by 2018.

### 2.2. Dataset

The 200 pelvic CT scans were used to develop a statistical shape model of the pelvis. The mean age of all patients was 56 ± 16 years. The distributions of age, weight, and height were comparable to the distributions found in the Dutch population [21,22]. The demographic information of the male and female groups is shown in Table 1. Body height and weight were statistically different between the two groups.

This study was conducted in accordance with the Strengthening of the Reporting of Observational Studies in Epidemiology (STROBE) guidelines and was performed in line with the Declaration of Helsinki [23].

### 2.3. Three-Dimensional Model Preparation

The left hemipelvis was segmented from the CT scans of the included patients to obtain a surface mesh of the left hemipelvis using Mimics (Materialise Mimics Medical 20.0, Leuven, Belgium) and 3D Slicer (version 4.11) [24]. The segmentation threshold was set to an HU value of 200. In some cases, an additional lower value of 80 was used to obtain complete segmentations. Possible cavities in the mesh were filled with an automatic operation in the software. If needed, the manual filling up of cavities was performed when these were still present after this automatic operation. All surface meshes were smoothed using 3-Matic Medical software (Materialise Mimics Medical 20.0, Leuven, Belgium) and 3D Slicer (version 4.11) [24]. To ensure a uniform distribution of points in each mesh, the meshes were remeshed with an isotropic edge length of 1.5 mm using a remesher function implemented in Matlab (MATLAB 2018b, The MathWorks, Inc., Natick, MA, USA) [25]. The hemipelves were aligned and registered to enable anatomical point-to-point correspondence, which is a requirement for a principal component analysis.

### 2.4. Principal Component Analysis and Statistical Shape Modeling

**Figure 1:**
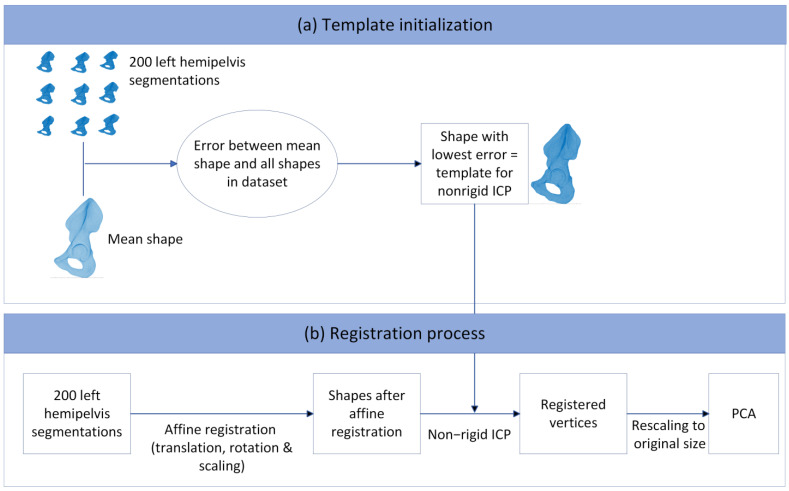
Registration process with the different steps that were taken, starting with the shapes in the dataset towards the principal component analysis. (**a**) Template initialization for the non-rigid ICP algorithm. This template was obtained by first calculating the mean shape from the shapes in the dataset. The patient in the dataset who resembles the mean shape best was chosen as the template. (**b**) Registration process. All the hemipelves in the dataset were initially translated to the same position, after which affine registration and non-rigid ICP were performed, resulting in registered vertices. These were rescaled to the original size and used as input for the principal component analysis (PCA).

Anatomical point-to-point correspondences between all the hemipelves were achieved by registering a template mesh to each individual hemipelvis using a non-rigid iterative closest point (ICP) algorithm as developed by Audenaert et al. (2019) [26,27]. As a first step, the translation of all the hemipelves was performed to ensure the alignment of the center of mass of each pelvis. Subsequently, the ICP algorithm registration was performed. The non-rigid ICP algorithm includes affine (translation, rotation, and scaling) and non-rigid registration steps. To avoid the bias that a random template can initiate, one initial registration was performed to obtain a mean shape. The error between each shape from the dataset and this mean shape was computed, and the mesh of the patient with the lowest error was used as the template mesh for the first registration iteration [8]. In total, three registration iterations were performed. After each iteration, the obtained mean shape was computed, and this shape was used as the template in the next iteration. Non-rigid registration consists of elastic deformation and ensures an anatomical point-to-point correspondence between all meshes, with the number of vertices defined by the template mesh (39,962 vertices, 79,924 faces). After both the affine and non-rigid registration steps, the obtained vertices were rescaled to their original size to correct for the scaling operation that was performed in the affine registration step. These registered vertices were used as input for a PCA, from which one SSM was created, based on the adjusted software work by Manu in Matlab (MATLAB 2018b, The MathWorks, Inc., Natick, MA, USA) [28]. Figure 1 shows the registration process, including the template initialization for the non-rigid IPC algorithm.

All individual left hemipelves in the dataset were then compared to this mean shape to determine their shape deviation from the mean shape. A principal component analysis of the resulting data then described the geometry of each left hemipelvis score for all principal components (PCs). The shape variance of each PC was visualized as a mesh of the ±3 standard deviations (SDs). The ±3 SD shapes describe 99.7% of the shape variation in the population in the dataset for that particular PC. The output of the SSM were PC scores, where each PC score corresponded to a standard deviation from the mean. The combined set of PC scores of each patient describes the unique shape features of that patient. After registration, we computed the mean female shape and the mean male shape by averaging the coordinates of all 100 female and 100 male patients, respectively.

### 2.5. Evaluation

The robustness of the SSM was quantitatively evaluated by calculating its compactness and generalization properties [29]. Compactness evaluates how many components are needed to describe a certain degree of variation in the dataset [29]. Generalization, or the reconstruction ability, describes the performance of the model to reconstruct a hemipelvis that has not been included in the dataset used to develop the SSM (the leave-one-out method) [29]. Generalizability therefore is a measure to check whether there is enough data in the model to represent the entire population that it was made for. One hemipelvis was removed from the dataset, and PCA was performed on the remaining hemipelvis segmentations in the dataset. The shape that was left out was reconstructed using the SSM, with a varying number of PCs included. The mean root mean square error (RMSE) for each hemipelvis was computed.

To provide an example of the use of an SSM in clinical practice, a reconstruction case was described. The case of a patient with a posterior wall fracture in the left hemipelvis was reconstructed by means of the SSM, and this reconstruction was compared to the intact contralateral right hemipelvis [30]. The SSM consists of the first few PCs that account for 90% of the shape variation. The RMSE between the reconstructed shape and the intact contralateral right hemipelvis was computed.

### 2.6. Statistical Analyses

The distributions of the first 15 PC scores were visually inspected by means of histograms and Q-Q plots to check for normality, since the first 15 PCs describe 90% of the total shape variation. Potential differences between the male and female subgroups of the first 15 PC scores of the SSM were assessed with a *t*-test [11].

## 3. Results

### 3.1. Shape Variation in the Pelvis

**Figure 2:**
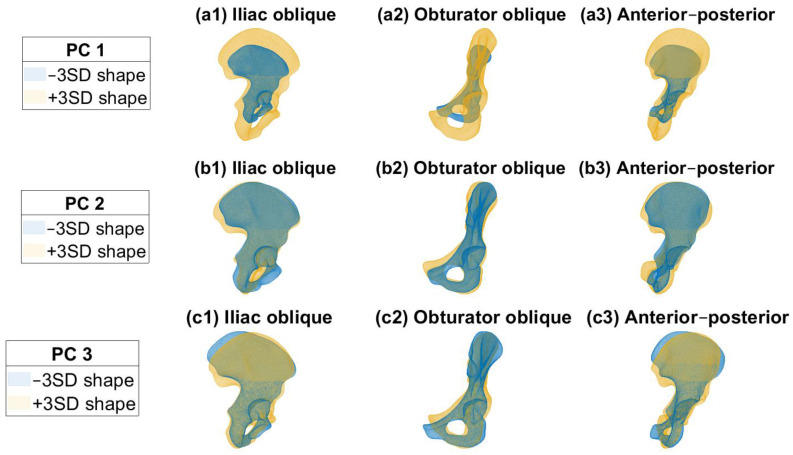
(**a1**–**a3**) Principal component (PC) 1 describes 68% of the total shape variation, mainly variation in size. (**b1**–**b3**) Principal component 2 describes 5% of the shape variation, mainly variation in curvature of the iliac wing and pubic region. (**c1**–**c3**) Principal component 3 describes 4% of the shape variation, mainly variation in the pubic region.

Each PC in the SSM describes a percentage of the total shape variation in the pelvis in descending order [9]. The first 15 PCs described 90% of the total shape variation in the pelvis. The compactness curve and generalization ability can be found in Figure S1 in the Supplementary Materials. Only the hemipelvis shapes were used as input for the model; the meaning of the resulting PC shapes is an interpretation of the user. In Figure 2, the first three PCs are shown, representing 68%, 5%, and 4% of the total shape variation. The first PC mainly describes the difference in size. The second PC mainly describes the difference in curvature of the iliac wing, whereas the third PC mainly describes the variation in the pubic rami. Videos of the first three PC shapes can be found in the Supplementary Materials. The reconstruction ability of the SSM resulted in a root mean square error (RMSE) of 1.58 (95% CI: 1.53–1.63) mm when 15 PCs were included in the SSM.

**Figure 3:**
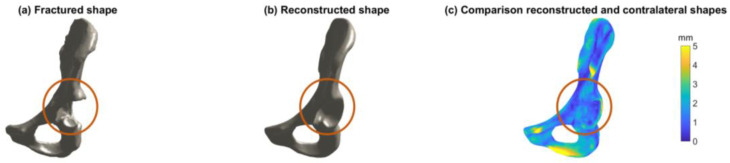
Example of a reconstruction case of an SSM in clinical practice. (**a**) Visualization of the right hemipelvis with a posterior wall fracture. (**b**) Visualization of the reconstructed shape, based on the SSM including the first 15 principal components (PCs). (**c**) Visualization of the difference between the reconstructed shape and the intact contralateral left hemipelvis by means of a heatmap. The color legend describes the RMSE (ranging from 0 to 5 mm) of the reconstructed shape and the intact contralateral hemipelvis. The mean RMSE was 1.6 ± 1.0 mm when the reconstructed shape was based on the SSM including the first 15 PCs.

Figure 3 shows an example of the use of an SSM in clinical practice, by means of a reconstruction case. Figure 3a shows the right hemipelvis of a patient with a posterior wall fracture. Figure 3b shows the reconstructed shape, consisting of the first 15 PCs describing 90% of the total shape variation in the dataset. Figure 3c shows a heatmap of the difference between the reconstructed shape and the intact contralateral left hemipelvis, with a mean RMSE of 1.6 ± 1.0 mm. The reconstruction ability of such an SSM can be used, for example, in virtual reconstruction for treatment planning when the contralateral hemipelvis is not available.

### 3.2. Male and Female Hemipelvis

**Figure 4:**
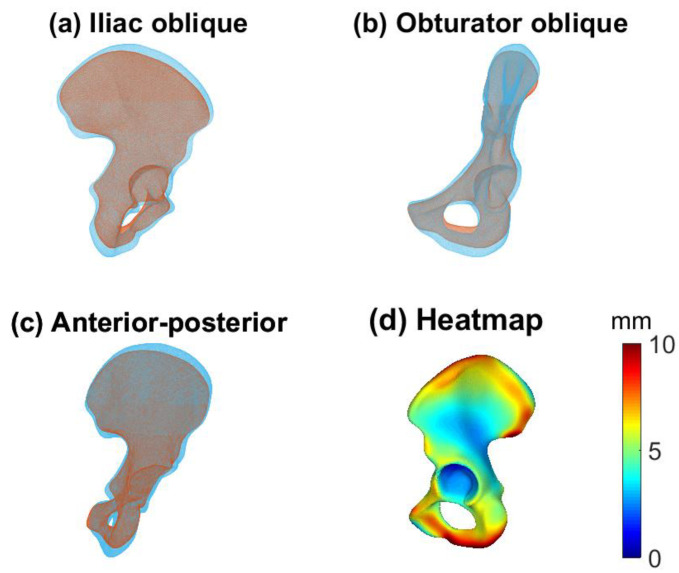
Mean male (light blue) and female (red) shapes for iliac oblique (**a**), obturator oblique (**b**), and anterior–posterior (AP) (**c**) pelvic views. (**d**) Differences between mean male and mean female shapes. The deviation of the mean male shape from the mean female shape is shown, projected onto the mean female shape. Red areas display a large difference, whereas blue areas display minor differences. Main differences are visible in the pubic area and in the iliac wing.

**Figure 5:**
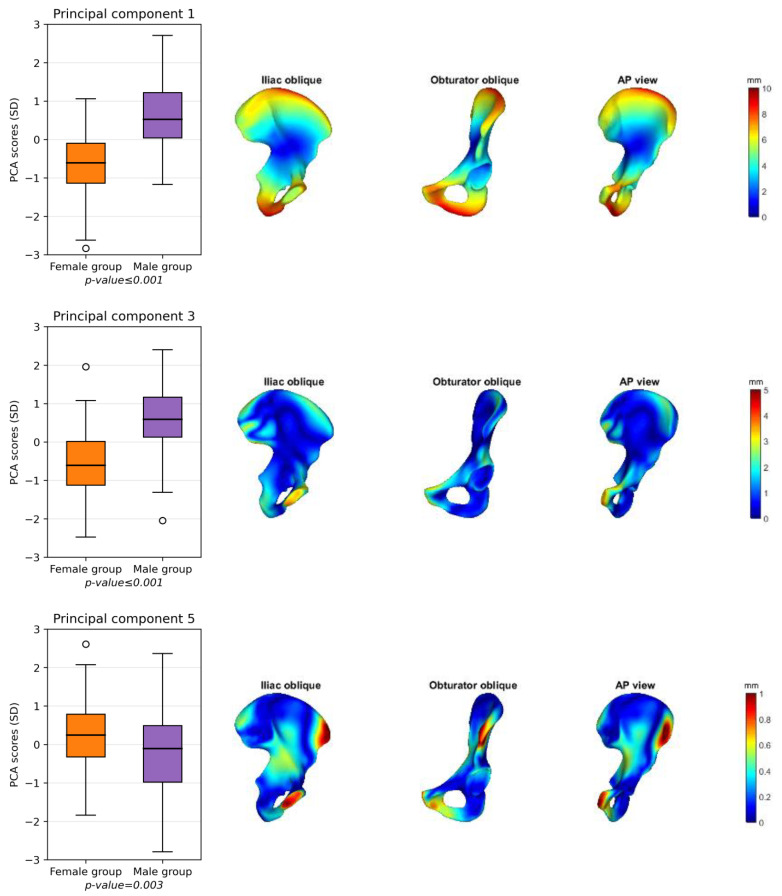

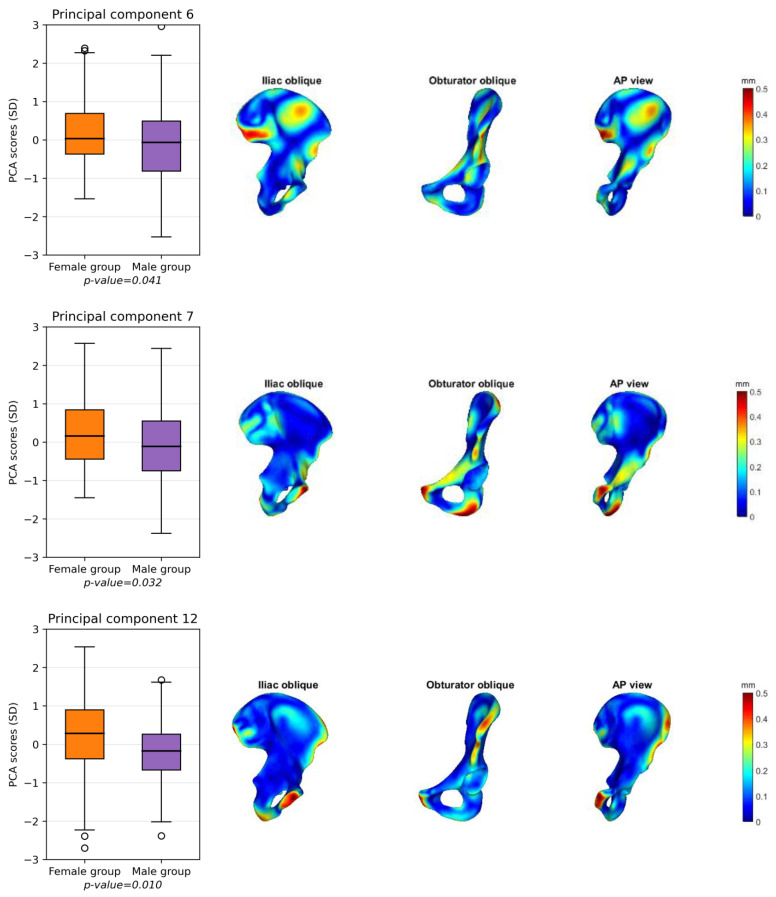
(**left**) Boxplots of the six principal components (PCs 1, 3, 5, 6, 7, and 12) that statistically differ between females (orange) and males (purple). (**right**) Projections of the shape deviation from the male pelvis compared to the female pelvis. For each PC, the difference between the mean male pelvis was projected onto the mean female shape in the iliac oblique, obturator oblique, and anterior–posterior (AP) views. Blue areas represent a small difference, and red areas represent a large difference. The main differences between male and female pelvis were found in the pubic area (PCs 1, 3, 5, 7, and 12) and iliac wing (PCs 1, 3, 5, 6, and 12).

Figure 4 shows the male (blue) and female (yellow) mean shapes in the iliac oblique (Figure 4a), obturator oblique (Figure 4b), and anterior–posterior (AP) (Figure 4c) pelvic views. Figure 4d shows the female mean shape with the deviation of the male mean shape projected onto it, resulting in a heatmap. The main differences are found along the iliac wing and the pubic region. PCs 1, 3, 5, 6, 7, and 12 are statistically different between the men and women (Figure 5). The heatmaps in Figure 5 show the differences between the mean female and mean male shapes for each specific PC, with the locations indicated in red indicating larger differences between the two shapes and the locations in blue indicating a smaller difference. The general mean shape, the female mean shape and the male mean shape can be found as STL files in the Supplementary Materials. 

## 4. Discussion

Our first objective was to develop an SSM of the hemipelvis and describe the pelvic shape variations in a Caucasian population. In conclusion, our newly developed SSM of the hemipelvis is able to reconstruct an aberrant hemipelvis. The 3D shape variation is described by the SSM by means of principal components (PCs), and 15 PCs were needed to describe 90% of the total shape variation. Our SSM demonstrated that the pre-dominant shape variations were related to size of the hemipelvis (e.g., PC1 represents size of the hemipelvis and describes 68% of the total shape variation), followed by differences in the curvature of the iliac wing (PC2 describes 5% of the variation) and differences in the pubic rami (PC3 describes 4% of the variation). This information is very relevant for implant companies. For them, using our SSM might be interesting in order to assess which sizes of pelvic implants should be produced to provide proper-fitting implants for most of the population.

Our second objective was to assess the potential differences in the shape variation between male and female hemipelves. Our SSM demonstrated that 6 out of the first 15 PCs differed between the male and female groups, mostly representing differences in the pubic area and the curvature of the iliac wing. These results are in line with the findings of Arand et al. (2019) and Ahrend et al. (2020) [19,20]. Arand et al. (2019) developed an SSM of 30 male and 20 female pelvic CT scans, whereas Ahrend et al. (2020) developed an SSM of generic Asian pelvic bones and showed differences in sex- and size-dependent anatomical variations within this population, consisting of 50 male and 50 female pelvic CT scans [19,20]. The patient population in our study is larger than that in previous studies and describes the differences in a Caucasian population. Since Audenaert et al. (2019) established that at least 200 training samples are needed to accurately cover population variance, our results are considered an accurate representation of the shape variation in a Dutch population [10].

Several applications of SSMs in the pelvis have been proposed recently. Krishna et al. (2022) compared the use of an SSM and contralateral mirroring to reconstruct the hemipelvis and found that contralateral mirroring had RMSE values comparable to those of the SSM, which included 64 hemipelves. In particular, the ischium and pubis regions had lower RMSE values when contralateral mirroring was used. This indicates that there is a high variability in these regions within a population, which we also found in this study [8]. Kriechling et al. (2022) performed a statistical shape model-based analysis of periacetabular osteotomies. The SSM was used to simulate the rotation of the osteotomized fragment to analyze the necessary translation as a function of the degree of correction [31]. Moreover, Meynen et al. (2022) used a statistical shape model to create a reconstruction of a disease or defect human anatomy. This reconstruction was evaluated and compared to three other models for acetabular defect classification [14].

In clinical practice, our SSM can be used as part of preoperative surgical planning. Our SSM is relevant to clinical practice, because the SSM is able to reconstruct a (aberrant or injured) hemipelvis of a newly admitted patient (Figure 3). In other words, the SSM is able to create a 3D model of how the patient’s pelvis should have looked before they sustained a pelvic injury or condition. The ability of the SSM to reconstruct a patient-specific shape from the mean shape resulted in a mean RMSE of 1.58 (95% CI: 1.53–1.63) mm when 15 PCs were included. In the previous literature on virtual pelvic reconstruction, errors ranging between 0.8 and 1.1 mm were found when 30 PCs were included [7,13,18]. These errors are lower than those found in our study, which could be due to the larger number of patients and, therefore, the larger number of PCs that were included in our study. Moreover, Verbeek et al. (2018) concluded that the critical CT cutoff value for total hip arthroplasty conversion was 5 mm for gap and 1 mm for step-off displacement [32]. This means that the margin of error of our SSM is limited, and, therefore, it could be relevant to clinical practice.

In future studies, such an SSM can be used for plate fitting by assessing and quantifying regions that are relevant for plate fitting in acetabular fractures. Moreover, an SSM can be used to generate a template for virtual reconstruction when the contralateral hemipelvis is not suitable (e.g., previously injured or in situ implants) [8,15]. An example of such a reconstruction case was provided in this study (Figure 3), using an SSM that included the first 15 PCs, accounting for 90% of the total shape variation in the dataset. This reconstruction example shows the clinical use of an SSM for virtual reconstruction in the case of unavailability of the contralateral side. A next step would be to assess shape variations in the regions of the pelvis that are important for plate fitting in pelvic fractures and to quantify these deviations from a standard plate. Moreover, the quantification of differences between male and female mean shapes might support the need for several sizes of acetabular plates, based on sex-related mean shapes. An SSM can also be implemented regarding other hip-related conditions, such as hip impingement, hip dysplasia, and hip preservation surgery (e.g., periacetabular osteotomy) [33]. Overall, this study provides a first impression where differences are found between male and female mean shapes.

The most important limitation is related to data acquisition. One limitation in the SSM development is that 77 patients were segmented with Mimics (Materialise Mimics Medical 20.0, Leuven, Belgium) and 123 patients with 3D Slicer (version 4.11). Even though two different segmentation software tools were used in this study, Mandolini et al. (2022) showed that these software tools have comparable geometrical and dimensional accuracies and do not affect our results [34]. Another limitation of the dataset is that we only included intact left hemipelves in the dataset. Bilateral symmetry of the pelvis was assumed, as was described in previous studies [35,36,37]. Moreover, this study aimed to represent the general Dutch population, thereby also including patients who suffer from non-bone hip-related medical conditions, for example, unexplained hip pain. It should be noted that our SSM was developed on CT data of adults, and, therefore, our SSM could not automatically be applied for the reconstruction of pediatric pelvises. Lastly, the quality of the CT scan and the segmentations might have influenced the error, since a smoother surface may result in a lower RMSE but might not be representative of the true deviation. Previously, (semi-)automatic segmentation tools of pelvic bones were developed that could be used in future studies to obtain segmentations [38,39].

## 5. Conclusions

In this study, a statistical shape model was developed of the hemipelvis of a general population. Our findings should be considered a first step in obtaining detailed knowledge about 3D region-specific pelvic shape variations. Anatomical shape variations were mostly related to differences in the size of the pelvis and most prominent in the regions of the iliac wing and pubic rami. Differences between the male and female pelvis were most pronounced in the iliac wing and pubic area. These regions are often subject to injuries, and, therefore, future applications of our newly developed SSM may focus on SSM-based semi-automatic virtual reconstructions of the pelvis and pelvic implant development.

## Figures and Tables

**Table 1:** Baseline characteristics.

	Total Group	Male Group (*n* = 100)	Female Group (*n* = 100)	*p*-Value
Age, years	56 ± 16	54 ± 17	57 ± 14	0.15
Height, cm	173 ± 10	180 ± 7	167 ± 7	**<0.001**
Weight, kg	81 ± 19	88 ± 18	74 ± 17	**<0.001**

Data are presented as mean ± standard deviation. *p*-values < 0.05 are displayed in bold.

## Data Availability

No new data were created or analyzed in this study. Data sharing is not applicable to this article.

## Supplementary Materials

The following supporting information can be downloaded at https://www.mdpi.com/article/10.3390/jcm12113767/s1, Figure S1: Compactness curve and generalization ability; Video S1: Principal component 1; Video S2: Principal component 2; Video S3; Principal component 3; General mean shape, female mean shape and male mean shape as STL files.
