# Supplementary figures and images for: Development of a Statistical Shape Model and Assessment of Anatomical Shape Variations in the Hemipelvis
Source: J Clin Med. 2023 May 30;12(11):3767. doi: 10.3390/jcm12113767 (PMC10253964; doi:10.3390/jcm12113767)

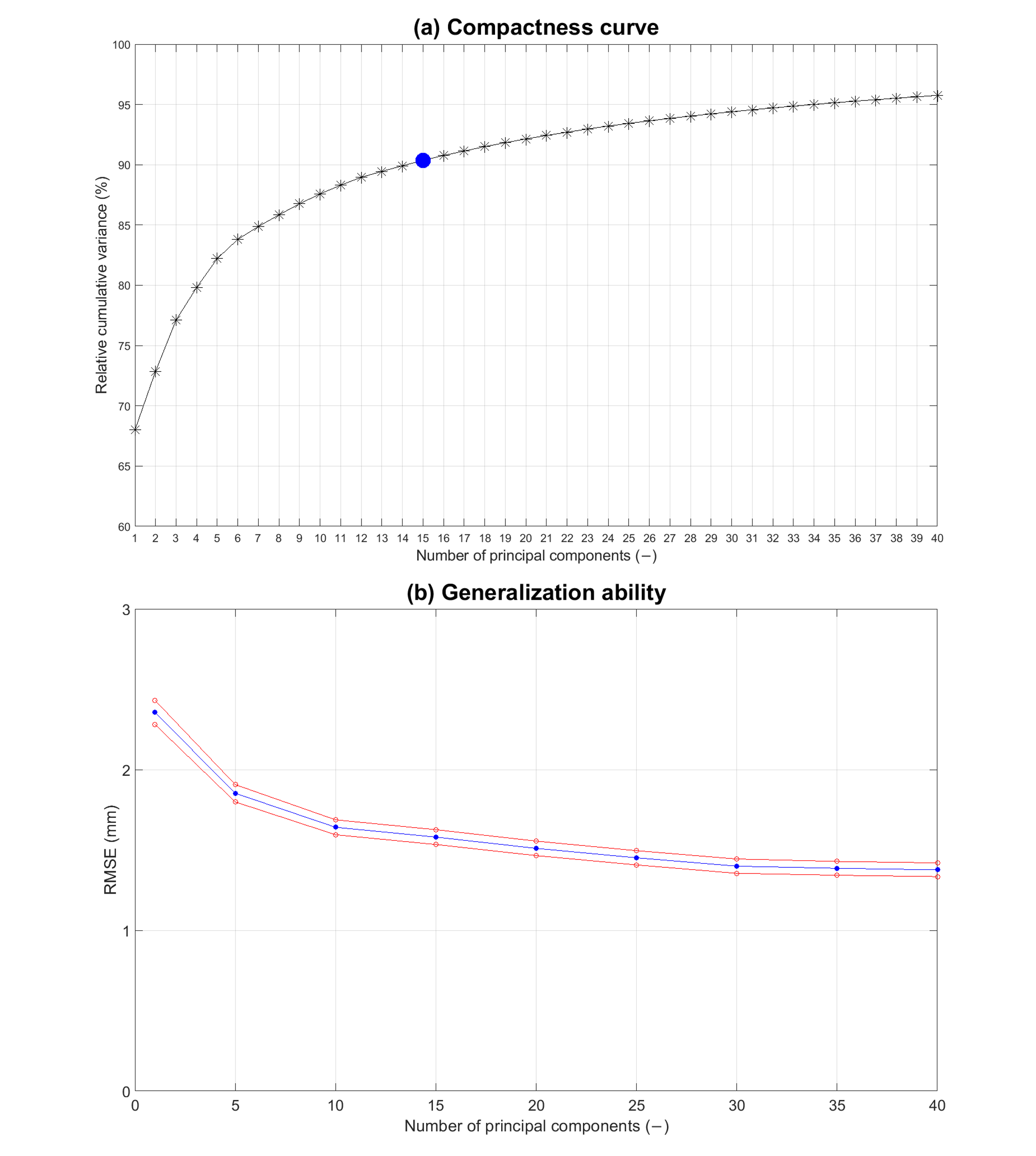

Supplement: Supplementary file 1 [file jcm-12-03767-s001.zip › Figure S1 Compactness curve and generalization ability.png]
